# Enhancing the Tribological Properties of Low-Density Polyethylene Using Hard Carbon Microfillers

**DOI:** 10.3390/ma17071536

**Published:** 2024-03-28

**Authors:** Samuel Solomon, Rachel Hall, Jibao He, Vijay John, Noshir Pesika

**Affiliations:** 1 Department of Chemical and Biomolecular Engineering, Tulane University, 6823 St. Charles Ave., New Orleans, LA 70118, USA; ssolomon2@tulane.edu (S.S.); vj@tulane.edu (V.J.); 2 New Product Development, Intralox LLC, 301 Plantation Rd., New Orleans, LA 70123, USA; rachel.hall@intralox.com; 3 Microscopy Laboratory, Tulane University, New Orleans, LA 70118, USA; jhe1@tulane.edu

**Keywords:** inter-molecular interactions, polymer composite, adhesion, deformation, shear stress, dry friction, surface roughness, shear force, real contact area, asperity junctions

## Abstract

The application of low-density polyethylene (LDPE) has been confined to packaging applications due to its inadequate mechanical and tribological characteristics. We propose enhancing LDPE by integrating hard carbon spheres (CSs) to improve its strength, frictional characteristics, and wear resistance. LDPE/CS composites were created by blending LDPE with varying CS amounts (0.5–8 wt.%). Analysis using scanning electron microscopy and Raman spectroscopy confirmed CS presence in the LDPE matrix, with X-ray diffraction showing no microstructural changes post-blending. Thermal characterization exhibited notable improvements in thermal stability (~4%) and crystallinity (~7%). Mechanical properties such as hardness and Young’s modulus were improved by up to 4% and 24%, respectively. Tribological studies on different composite samples with varying surface roughness under various load and speed conditions revealed the critical role of surface roughness in reducing friction by decreasing real contact area and adhesive interactions between asperities. Increased load and speed amplified shear stress on asperities, possibly leading to deformation and failure. Notably, integrating CSs into LDPE, starting at 1 wt.%, effectively reduced friction and wear. The composite with the highest loading (8 wt.%) displayed the most significant tribological enhancement, achieving a remarkable 75% friction reduction and a substantial 78% wear reduction.

## 1. Introduction

Low-density polyethylene (LDPE) finds extensive use in consumer and packaging industries owing to its flexibility, cost-effectiveness, and resistance to moisture and chemicals. Yet, within the polyethylene family, LDPE is the softest variant, limiting its suitability for many engineering applications. Its high level of chain branching obstructs the formation of crosslinked, interconnected networks, which means that the polymer has a less compact molecular structure with a low density [1]. Consequently, LDPE exhibits inferior mechanical and thermal properties, including hardness, tensile strength, and melting point, as well as inadequate resistance to friction and wear due to adhesion and deformation. 

Incorporating filler materials into polymer matrices is a widely employed strategy, renowned for its ability to improve both the cost-effectiveness and performance of composite materials. Notably, these particles or fillers have been shown to enhance various properties of polymers, including electrical, mechanical, and tribological characteristics [2,3,4,5,6,7,8,9,10,11,12,13]. In keeping with this approach, recently many researchers have explored a diverse array of inorganic and organic filler materials to bolster the mechanical and tribological characteristics of LDPE. Examples include iron scale (Fe_2_O_3_) [14], marble dust [15], rice husk ash (RHA) and silica sand [16,17], Al_2_O_3_/SiC [18], biochar [19], Al_2_O_3_ + TiO_2_ [20], graphene [21], lignin [22], montmorillonite (MMT) [23], blast furnace dust [24], and coconut husk powder/coir [3], etc.

Despite the wealth of research on enhancing the mechanical and tribological properties of polymer composites, the utilization of carbon spheres (CSs) remains largely unexplored in the literature. CSs possess exceptional characteristics, including ease of synthesis, spherical morphology, and favorable surface and mechanical properties [25,26,27,28]. These unique attributes render CSs potentially superior as polymer fillers compared to other commonly studied materials. Notably, the spherical shape of CSs ensures optimal packing density, uniform stress distribution, and eliminates potential stress points that may lead to composite failure [29]. Furthermore, the hydrophobic surface chemistry of CSs facilitates interaction with nonpolar LDPE, ensuring excellent dispersibility without requiring surface modification. Additionally, CSs boast superior mechanical properties, with a tensile strength of up to 8 GPa, and exhibit stability under a wide range of thermal and chemical conditions [25,30]. Consequently, the application of CSs as fillers in polymers to enhance mechanical and tribological properties holds great promise. This study seeks to explore this promising avenue.

Polymer friction and wear are influenced by both molecular and mechanical components [31]. The molecular aspect arises from adhesion/cohesive forces among polymers, occurring at asperity junctions that are facilitated by van der Waals and electrostatic forces between contacting surfaces [32]. This polymer adhesion contributes significantly to friction in polymers and can be lessened by the addition of fillers to the matrix of the polymer [33]. The mechanical component, on the other hand, is influenced by parameters such as applied load, contact area, sliding speed, and surface roughness of the material. Few studies have investigated how these parameters affect friction and wear in polymer composites. Soni et al. examined the impact of surface topography on the tribological performance of polymeric composites, including LDPE, and sought to understand the correlation between wear behavior and material properties [17]. They observed that the wear rates of the composites were influenced by both the matrix and surface roughness. The wear rate exhibited an irregular increase with surface roughness due to changes in the coefficient of friction during relative motion. 

Additionally, Tian et al. conducted experiments on multi-fillers reinforced epoxy composites (MFREC) under dry and wet conditions to investigate the effects of applied loads and sliding speed on friction behaviors and wear mechanisms [34]. They observed that the effect of loads on the coefficient of friction was realized by changes in the real contact area. The change in contact area was determined by the contact state (elastic/viscoelastic or plastic/viscoplastic) which affected the coefficient of friction. Conversely, increased speed led to a higher coefficient of friction due to the uncoordinated deformation of surface and subsurface, thereby increasing the likelihood of material surface loss. Despite this research, there remains limited understanding of the correlation between the molecular (adhesion) and mechanical components (load, speed, surface roughness) and their impact on friction and wear behavior in polymer-filled composites.

This study pioneers the utilization of carbon spheres (CSs) in low-density polyethylene (LDPE) to enhance friction and wear properties. It delves into the intricate interplay between adhesion, influenced by operational factors and material characteristics such as load, speed, and surface roughness, and its impact on friction and wear. LDPE/CS composites, varying in particle loadings, underwent comprehensive mechanical and tribological assessments under diverse speed, load, and material roughness conditions. The introduction of CS fillers in LDPE was found to modulate both molecular and mechanical contributors to friction and wear. The innovative LDPE/CS composites developed herein hold promise for broad engineering applications spanning biomedical, automotive, industrial, and aerospace sectors. Furthermore, the newfound insights garnered from this study pave the way for the design and development of advanced polymer-filler composite materials with enhanced performance characteristics in dry conditions.

## 2. Materials and Methods

### 2.1. Preparation and Characterization of LDPE/CS

Carbon spheres were synthesized through the hydrothermal carbonization process [27] from a glucose precursor. A 0.3 M glucose solution was made by adequately dissolving some amounts of glucose (D-(+)-Glucose, Sigma-Aldrich, St. Louis, MO, USA; ≥99.5% HPLC grade) in deionized (DI) water. The glucose solution was placed in an airtight autoclave reactor and subjected to heating in an oven at 190 °C for 4.5 h. Subsequently, after cooling, the suspension underwent multiple washes with water and ethanol followed by centrifugation to collect the carbon particles. The synthesized carbon was then dried in an oven at 60 °C overnight. Furthermore, the obtained carbon underwent pyrolysis in a furnace at 1000 °C for 10 h within an ultrapure nitrogen environment to yield hard carbon spheres [35,36]. The as-prepared particles were imaged to determine size and morphology using scanning electron microscopy (SEM, Hitachi S-4800 FE-SEM, Yongin-si, Republic of Korea) operated at 20 KV.

The preparation of the composite samples involved the addition of carbon spheres (CSs) to LDPE powder (polyethylene powder, low-density 500 microns, Alfa Aesar, Heysham, UK) in precise amounts. The two materials were mixed thoroughly in dry form and then poured into a mold and placed in an oven, operated at 190 °C. This powdery mixture was allowed to remain for 10–15 min in the oven to enable it to melt completely within the mold. Subsequently, the molten blend was removed from the oven, pressed down, and left to cool down under ambient conditions. The composite sample was then separated from the mold in solid form. Composites with varying CS loadings were prepared, ranging from 0.25 to 8 percent by weight (wt.%) (refer to Table 1 for sample designation and composition). Diverse mold shapes were designed and machined to fabricate samples suitable for friction/wear and tensile tests.

SEM was also employed to observe the matrix of virgin LDPE and one of the composite samples (LDPE/CS_5). This time, it was operated at a lower voltage of 3 KV to prevent a charging effect. To image the composite, a tiny specimen was made and then sonicated in an acetone bath to expose some particles. Prior to imaging, a fine layer of carbon was coated on the samples to prevent charging and to optimize image quality. X-ray diffraction (XRD) was utilized to analyze the structural characteristics of the samples. A Rigaku Miniflex 600 diffractometer (Rigaku Corporation, Tokyo, Japan), functioning at 40 kV and 15 mA, was used to scan the samples within a range of 2Ɵ (4 to 60°) at a scanning speed of 2.4°/min.

Raman spectroscopy, performed using the WITec alpha 300 Confocal Raman imaging microscope (WITec, Ulm, Germany), provided further insights into the composite samples’ characteristics. Three samples (CS, pristine LDPE, and LDPE/CS_6) were scanned utilizing a laser power of 9.9 mW with an excitation wavelength of 532 nm. The laser power was carefully selected to avoid degradation of the polymer sample. The objective magnification was 50×. The scanning process for CS involved obtaining a single point spectrum with 200 accumulations and an integration time of 1 s. Conversely, the LDPE and LDPE/CS_6 samples were imaged using a line scan (45.5 µm in length), with 100 accumulations and an integration time of 0.5 s.

### 2.2. Thermal Characterization

Three specimens for each sample group underwent Differential Scanning Calorimetry (DSC, New Castle, DE, USA) to examine alterations in melting temperature, crystallinity, and crystallization temperature due to the presence of CSs in the polymer. The experiments were conducted under a nitrogen atmosphere flowing at a rate of 50 mL/min. Each sample, weighing between 3 mg and 5 mg, underwent three cycles: heating to 180 °C at a ramp rate of 10 °C/min, cooling to −90 °C at a rate of 5 °C/min, and then reheating to 180 °C at a ramp rate of 10 °C/min, with 5-min isothermal holds between each cycle. The degree of crystallinity (X_C_) of the samples was calculated using Equation (1) [37].

(1) $$X_{c}=\frac{{∆H}_{f}(T_{m})}{{∆H}_{f}^{0}(T_{m}^{0})}\times 100$$

The degree of crystallinity (X_C_) was calculated using the enthalpy of fusion, ∆H_f_ measured at the melting point, T_m_ (derived from DSC plot), and the enthalpy of fusion of the entirely crystalline polymer, ∆H_f_^0^ measured at the equilibrium melting point, T_m_^0^. For polyethylene, ∆H_f_^0^ is 290 J/g [38]. For each sample group, the reported X_C_ values are an average of the three specimens measured.

To assess the thermal stability of the composite samples in contrast to the pristine polymer, Thermogravimetric Analysis (TGA) was conducted using TGA Q500 equipment (TA Instruments, New Castle, DE, USA). The tests were conducted under a nitrogen flow to prevent oxidation of the CS particles in the sample. The sample weighed between 10 mg and 15 mg, and the heating process ranged from room temperature to 600 °C at a heating rate of 5 °C/min.

### 2.3. Mechanical Characterization

Tensile tests were conducted on three replicates per sample group according to a modified version of ASTM D638 [39] using a servohydraulic Universal Testing Machine (Instron 8801, Norwood, MA, USA) and dynamic axial clip-on extensometer (Instron 2620 Series). The coupons used were produced from a mold 110 mm in length, 20 mm in width, and 3.5 mm in thickness. The narrow region was 40 mm long and 13.5 mm wide. Thickness and width of the narrow region were measured for each coupon before testing to calculate engineering stress. The tests were carried out under ambient conditions with a crosshead speed of 1.5 inches/min (100% strain/min). The extensometer had a gauge length of 25 mm with a 50% strain limit. Each coupon was pulled until failure or until the extensometer limit was reached. Engineering stress and strain were recorded for each specimen. Young’s modulus, maximum stress, and strain at maximum stress were obtained from the tests and the average of the three replicates for each sample group were reported for this study. 

The Guth equation [40] (Equation (2)) was used to predict the theoretical values of the modulus of filled polymers:

(2) $$G_{f}=G_{0}(1+2.5\varphi +14.1\varphi^{2})$$

Here, G_f_ and G_0_ represent the modulus of the filled and unfilled polymer material, respectively, and φ denotes the volume fraction of the filler. The experimental modulus values were validated by comparing them to the theoretical values predicted by the Guth equation.

Additionally, a Shore Durometer was employed to measure the hardness (Shore D) of the samples, with the reported values being an average derived from 4 points measured from each of the 3 specimens in a sample group.

### 2.4. Tribology Experiments

The friction and wear assessments were conducted using a tribometer (CETR universal materials tester, Multi-Specimen Test System, Billerica, MA, USA) equipped with a DFH-20 force sensor (Billerica, MA, USA) ranging from 0.2 kg to 20 kg. A rotational pin-on-disk setup according to a modified ASTM G99-17 [41] was used for the tribology tests. The top surface or pin was a spherical high-density polyethylene (HDPE) pin (diameter = 3.5 mm and surface roughness, R_a_ = ~225 nm) for the friction test, and a flat stainless steel (SS) pin (304 grade; diameter = 3.5 mm and R_a_ = ~180 nm) for the wear runs. The bottom surface or disks were LDPE and the LDPE/CS composite samples shaped in disk form. The counterfaces (i.e., HDPE and SS) were intentionally chosen to simulate the polymer–polymer and polymer–metal interactions found in practical applications. HDPE was selected because it is a stronger material and can interact with LDPE. The composite samples (i.e., the discs) utilized in the friction experiments were prepared with different surface finishes using an ECOMET IV grinder/polisher (Buehler, Salem, MA, USA) and SiC polish papers (180 grit and 600 grit, Allied High Tech Products, Inc., Rancho Dominguez, CA, USA). These preparations resulted in samples with “rough” (R_a_ = 1.67 µm ± 0.21 µm) and “smooth” (R_a_ = 181 nm ± 5 nm) surface profiles. The friction experiment was designed under varying load (5 N/20 N) and speed (10 RPM/100 RPM, equivalent to ~8.4/84 mm/s) conditions. 

To study the impact of crucial parameters like normal load, sliding speed, and surface roughness on the friction coefficient of the composite materials, various tests were conducted according to a robust experimental design found in Table 2. The friction coefficients (which is the friction force divided by the applied load) were derived from the experiments and used in plotting the graphs. 

A similar set up was used for the wear tests. The major difference is the use of a flat-faced cylindrical stainless steel pin as the top shearing surface to maintain consistent pressure throughout the test. Since SS is significantly harder than LDPE, we assumed that wear would occur only in the bottom composite samples and characterize these only. Moreover, the test parameters were different—with an applied load, rotational speed, and time of 50 N, 100 RPM, and 6 h, respectively. The sample used for wear runs had a surface roughness value of 181 nm (smooth). The wear profile of the samples post wear tests was analyzed utilizing an optical microscope (magnification, 10×), while the wear depth and profile were evaluated using a confocal microscope (Rtec instruments, San Jose, CA, USA) with a BF 20× setting. To determine wear depth, multiple observations (3+) were taken from distinct areas across the wear track, and the reported wear depth values represent the averages of these measurements. All experiments (friction and wear) were conducted under dry conditions.

## 3. Results and Discussion

### 3.1. Morphology and Structural Characterization of LDPE/CS

**Figure 1:**
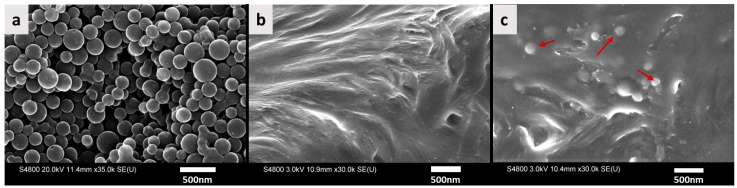
SEM image of (**a**) CS particles, (**b**) pristine LDPE matrix, (**c**) LDPE/CS_5 (LDPE + 4 wt.% CS). The red arrows show the location of some CS particles.

Figure 1a–c display the SEM images of CS particles, and the matrices of the pristine polymer and LDPE/CS_5 (4 wt.%) composite. The particle size ranged from approximately 150 nm to 350 nm. Notably, Figure 1c shows particles entirely entrapped within the polymer matrix (i.e., no void space or cracks present between the particles and the polymer), indicating favorable interaction between the particle and polymer (see Figure S1 for further SEM images).

Regarding the structure of samples, the virgin polymer and all composite samples were found to exhibit similar XRD spectra, suggesting that the CS does not induce alterations in the LDPE polymer structure. Further details on the XRD plots can be found in Figure S2a,b in the supporting document.

**Figure 2:**
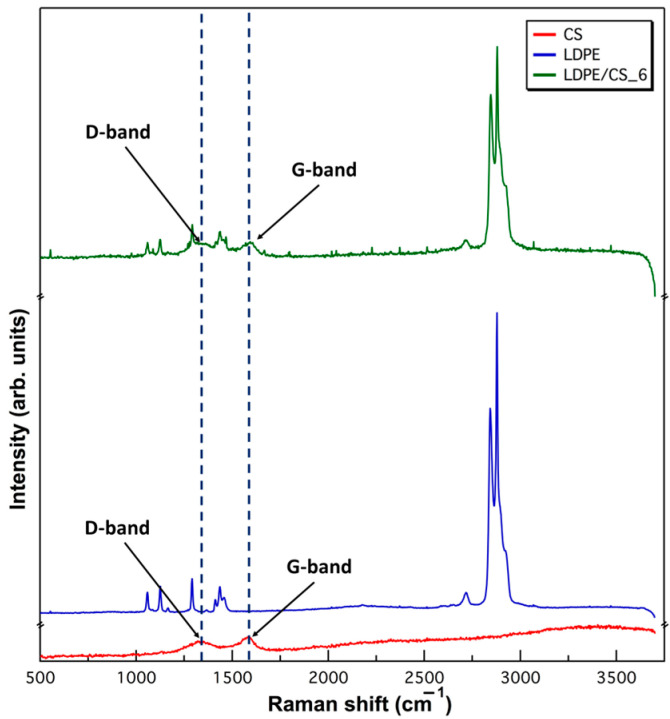
Raman spectra of CS, LDPE, and LDPE/CS_6 (LDPE + 8 wt.% CS).

Raman spectroscopy was applied to characterize the composites because amorphous carbon typically has Raman responses at 1355 cm^−1^ (D-band) and 1575 cm^−1^ (G-band), as reported in other work [42]. Therefore, the presence of CSs or lack thereof would easily be detected via Raman imaging. In Figure 2, the Raman spectra of CS, LDPE, and LDPE/CS_6 (8 wt.%) are depicted. The observations revealed the presence of D- and G-bands in both CS and LDPE/CS_6 samples, indicating the presence of CSs within the LDPE matrix of the composite.

### 3.2. Thermal Characterization

**Figure 3:**
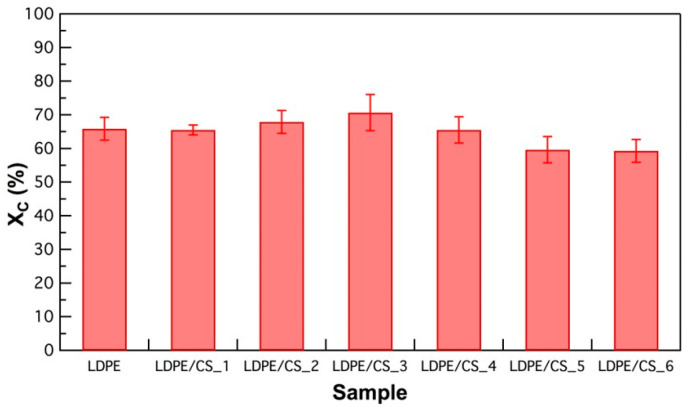
Plot showing the degree of crystallinity in samples with different CS loadings.

The crystallinity, X_C_ (in %), of both the pristine polymer and all composite concentrations derived from the DSC experiments according to Equation (1) is depicted in Figure 3. The X_C_ value exhibited a marginal increase with increasing CS loading, up to the 1 wt.% composite, before experiencing a slight reduction with further increase in CS content. This trend could suggest that, at lower concentrations, the particles might serve as nucleation sites for polymer crystallization [43]. As indicated in Table S1, an increase in particle loading resulted in a slight elevation in the crystallization temperature of the composite compared to the pristine polymer, signifying earlier crystallization at higher temperatures. However, at concentrations greater than 1 wt.%, the particles may have impeded crystal growth and inhibited the mobility of polymer chains, hence the marginal decrease in X_C_. This reduction in X_C_ at higher concentrations agrees with the study by Iji et al., who observed a decrease in crystallinity at 30 wt.% filler concentration [44].

**Figure 4:**
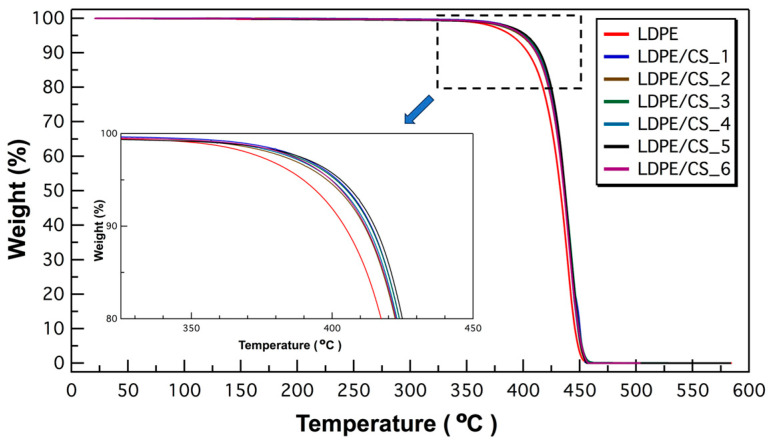
Plot of TGA result for LDPE and LDPE/CS composite samples. The inset shows a magnified view of the TGA data.

The thermogravimetric analyses (TGAs) revealed an enhancement in the thermal stability of the LDPE matrix due to the presence of particles. As depicted in the normalized TGA plots in Figure 4, a delay in the onset degradation (quantified by Td_5_, the temperature at which the sample loses 5% of its weight) is evident in an increase from 391.2 °C for LDPE to 405.7 °C for LDPE/CS_3. Additionally, the temperature at 50% weight loss (Td_50_) exhibited an elevation from 433.7 °C for LDPE to 438.4 °C for LDPE/CS_5 (details in Table S2). Previous research has established a direct correlation between a polymer’s thermal stability and its crystallinity [45]. This relationship might explain why LDPE/CS_3, with the highest crystallinity, also displayed the highest thermal stability, indicated by the elevated degradation temperature at 5% weight loss (Td_5_). Furthermore, the improved thermal stability could be attributed to enhanced interfacial interactions between the CS surface and the LDPE matrix, potentially increasing the thermal breakdown activation energy [21]. In previous studies on LDPE composite, improved thermal stability is also attributed to a reduction in chain motion and thermal vibration, and the hindrance effect to the presence of filler material [15,19].

### 3.3. Mechanical Characterization

**Figure 5:**
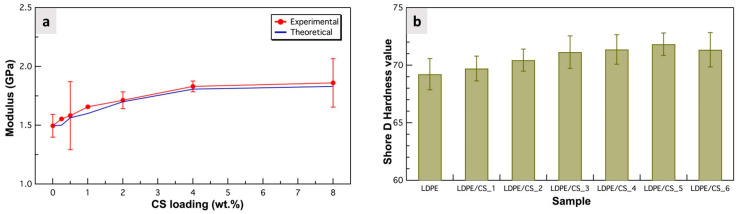
(**a**) Plot showing the experimental vs. theoretical Young’s modulus, and (**b**) bar chart of Shore D hardness of samples as a function of CS loading.

Mechanical tests were critical for evaluating the effect of the particles on the polymer’s mechanical properties. The modulus of the composites notably increased with the concentration of particles from LDPE/CS_1 (0.25 wt.% CS) to LDPE/CS_5 (4 wt.% CS) (Figure 5a). This upsurge is attributed to the greater stiffness and strength of the particles in comparison to pristine LDPE, contributing to a stiffer composite that is more resistant to deformation. However, beyond this concentration, particularly at 4 wt.%, the modulus remained almost constant. Figure 5a shows that the average Young’s modulus values from the experimental data are in close alignment with the theoretical values from the Guth equation. This suggests excellent interfacial adhesion between the CS particles and the LDPE matrix. Inclusion of CSs yielded an enhancement in the modulus of up to ~22% and ~24% (as observed in LDPE/CS_5 and LDPE/CS_6, respectively) in comparison to the virgin polymer. This improvement is higher than reported values in the literature (5.38% and 18% for the best LDPE/Al_2_O_3_/SiC and LDPE/ biochar composite, respectively) [18,19]. Table S3 provides additional mechanical properties of the samples, including the stress and strain values at maximum force.

The Shore D hardness values (Figure 5b) were also found to improve incrementally with increasing CS filler concentration up to the 4 wt.% (with a 4% increase compared to pristine LDPE). This improvement could be attributed to the hardening effect of the hard CSs and the decrease in interparticle distance on the surface as particle content increased within the polymer. Such improvement in hardness with filler concentration is reported by other studies and attributed to the reinforcing properties of hard fillers [15,20]. Al-Jumali et al. found the hardness of LDPE/MMT to increase compared to virgin LDPE due to enhanced interactions and crosslinking between the molecular chains of the composite [46]. In this work, the improved particle packing density at the surface of the composites contributes to greater surface resistance, thereby minimizing plastic deformation upon the application of a load. The hardness value of LDPE/CS_6 was marginally reduced compared to that of LDPE/CS_5. A possible explanation for this is that with higher concentration there may be more unfilled regions than expected due to particle agglomeration in certain spots, causing a slight reduction in surface resistance to load in those areas.

### 3.4. Tribological Studies

#### 3.4.1. Friction Study on LDPE/CS Composites

1. Dry friction model around asperity contact

**Figure 6:**
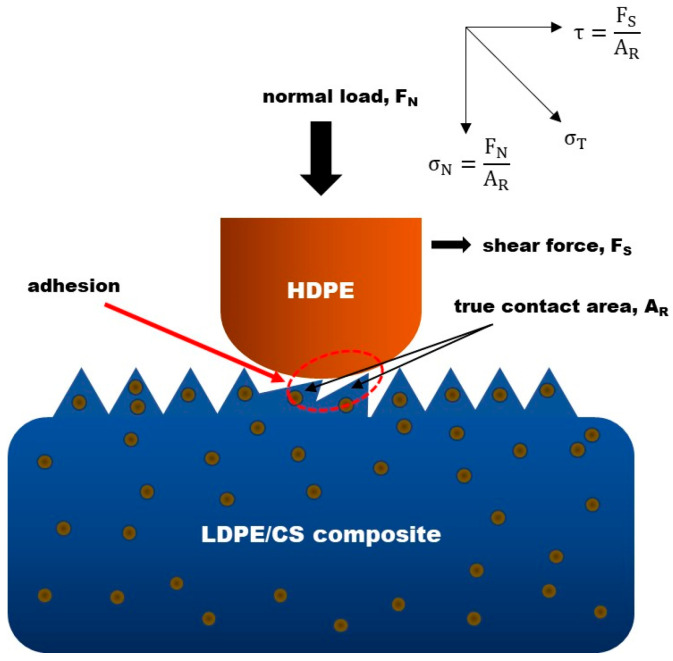
Schematic model showing the forces that act on polymer asperities during dry contact. The red dashed circle spotlights the true area of contact where adhesion takes place.

To provide a mechanistic explanation of friction and wear behavior, a model is developed around the point of contact at the microscopic level. Figure 6 depicts contact between two surfaces. A simplistic approach is taken by assuming that the top surface has no roughness. This would allow the model to be derived based on the bottom composite sample. Based on the molecular–mechanical theory, the total friction force, $F_{total}$, consists of both molecular and mechanical components [31], which can be expressed as follows:

(3) $$F_{total}=F_{molecular}+F_{mechanical}$$

$F_{molecular}$ originates from adhesion/cohesive forces among polymers, occurring at the point of contact between the interacting surfaces, while the mechanical component of friction, $F_{mechanical}$, arises from load, shearing speed, and surface roughness influence. True contact occurs between the mating asperities (the size of which depends on the surface roughness), therefore the real contact area is denoted by A_R_ [47]. The asperities experience a normal force F_N_ from the load and a shear force F_S_ in the sliding direction. Therefore, the total force, F_T_ (equivalent to $F_{mechanical}$), exerted on the asperities is the resultant of both force components. That is,

(4) $$F_{T}^{2}=F_{N}^{2}+F_{S}^{2}$$

Given that these forces act on asperities with contact area A_R_, the stress experienced by them becomes,

(5) $${\left(\frac{F_{T}}{A_{R}}\right)}^{2}={\left(\frac{F_{N}}{A_{R}}\right)}^{2}+{\left(\frac{F_{S}}{A_{R}}\right)}^{2}$$

or,

(6) $$\sigma_{T}^{2}=\sigma_{N}^{2}+\tau^{2}$$

where $\sigma_{T}$ is the total stress on each asperity, which is the resultant of the normal stress $\sigma_{N}$ and shear stress τ components. This model suggests that, in addition to the significant adhesive interactions with the counterface, the asperities on the composite sample also endure substantial forces induced by both the applied load and shearing speed. These forces have the potential to cause deformation and eventual failure of the asperities.

2. Influence of load and speed vs. particle concentration on friction coefficient

**Figure 7:**
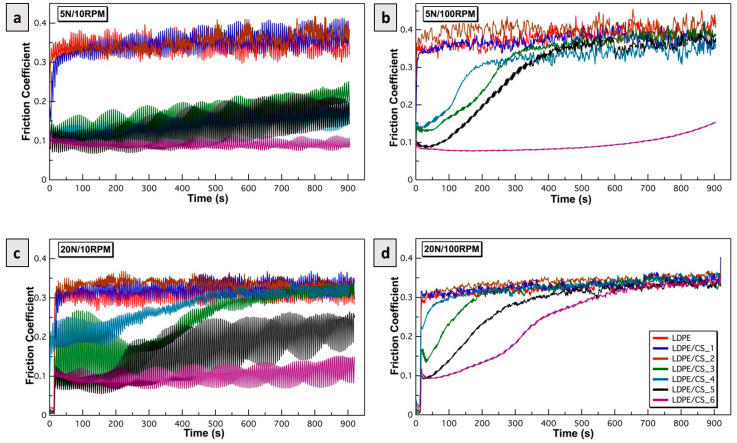
Plot of friction coefficient between smooth LDPE, LDPE/CS samples (R_a_ = 181 nm) against HDPE as a function of time under (**a**) 5 N/10 RPM, (**b**) 5 N/100 RPM, (**c**) 20 N/10 RPM, and (**d**) 20 N/100 RPM.

Figure 7a–d are plots of the friction coefficients versus time for the smooth samples at various combinations of load and speed. The friction model developed above was applied to accurately delineate the friction mechanism behind these results. The results in Figure 7 reveal that the presence of CSs in the polymer was only effective in lowering the friction coefficients after a critical concentration of 1 wt.% was reached. Generally, friction was observed to decrease with increasing CS contents. Across all of the load/speed conditions experimented under, the sample with the highest CS loading (LDPE/CS_6) had the lowest friction coefficients. Below, an elucidation of the friction mechanisms based on the developed model is provided. 

Under low loads and speeds (5 N/10 RPM—Figure 7a), samples with minimal or no particle content (LDPE, LDPE/CS_1, and LDPE/CS_2) exhibited notably high friction due to dominant adhesive forces. This is likely because the particle distribution in the polymer was too sparse to break the adhesion at the asperity junctions. However, starting at a particle concentration of 1 wt.% (LDPE/CS_3), friction began to decrease owing to the presence of CS particles, effectively mitigating the polymer–polymer interaction. LDPE/CS_6 demonstrated the most promising performance in breaking this interaction.

At low load/higher speeds (5 N/100 RPM—Figure 7b), LDPE, LDPE/CS_1, and LDPE/CS_2 maintained high friction levels due to both adhesive effects and debris formation from the shearing and fracturing of asperities. Initially, the LDPE/CS_3, LDPE/CS_4, and LDPE/CS_5 composites benefited from the higher particle content, which aided in inhibiting adhesion. However, the larger shear forces due to increased speed subsequently fractured mating asperities at varying intervals, potentially creating debris and elevating friction. Only LDPE/CS_6 exhibited adequate strength and rigidity against the $\sigma_{T}$ exerted by the counter body, resulting in the lowest friction coefficient throughout the study duration.

Under low speed/high load conditions (20 N/10 RPM—Figure 7c), the observed trends were similar to that of Figure 7b. The increased load increased the value of $\sigma_{T}$, resulting in instant failure at asperity junctions for the pristine polymer and composites of low concentrations. Whereas, with increasing particle loading, (i.e., LDPE/CS_5 and LDPE/CS_6) there was an improved resistance to adhesion and deformation due to the normal and shear load. 

At high speeds and loads (20 N/100 RPM—Figure 7d), the friction coefficients in all samples were dominated by normal/shear forces. The asperities of samples with lower CS concentrations succumbed more swiftly, while those with higher concentrations gradually succumbed to immense stress on their asperities. The total stress, $\sigma_{T}$, was high enough to impact LDPE/CS_6 over time, leading to the fracturing of its surface asperities. In Figure 7d, all samples demonstrated similar friction coefficients after approximately 700 s, potentially signifying the point where all asperities were removed, resulting in increased surface–surface interactions or a “sticky” effect (refer to Figure S3).

3. Influence of roughness vs. particle concentration on friction coefficient

**Figure 8:**
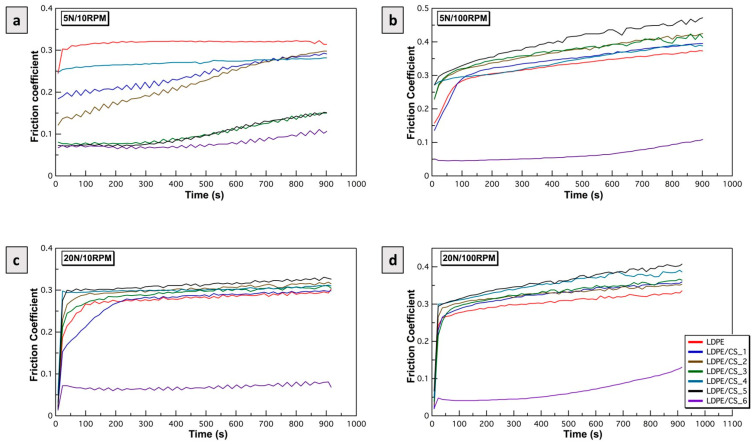
Plot of friction coefficient between rough LDPE and LDPE/CS samples (R_a_ = 1670 nm) against HDPE as a function of time under (**a**) 5 N/10 RPM, (**b**) 5 N/100 RPM, (**c**) 20 N/10 RPM, and (**d**) 20 N/100 RPM.

The results of the friction experiments for rougher samples (R_a_ = 1670 nm) are presented in Figure 8. Figure 8a–d are plots showing the friction coefficients over time for all samples under 5 N/10 RPM, 5 N/100 RPM, 20 N/10 RPM, and 20 N/100 RPM, respectively. Under low loads and speeds (5 N/10 RPM—Figure 8a), comparable trends to those in Figure 7a were observed. However, the friction coefficients in this scenario were lower for most samples due to the contribution from the surface roughness. At higher R_a_ values, the aspect ratio of asperities is higher, and the real area of contact (A_R_) at the asperity junction is decreased resulting in reduced surface interaction and adhesion. 

At higher loads or speeds or both (5 N/100 RPM, 20 N/10 RPM, 20 N/100 RPM—Figure 8b–d), the effects were similar across most rough samples except for LDPE/CS_6. Under these conditions, in which increased shear and normal stress were induced, the high aspect ratio asperities were swiftly deformed and fractured. Larger amounts of particles in the debris formed and were observed in samples with higher particle loading, which acted as third bodies that ploughed the surface and led to increased friction during shearing. Consequently, the friction coefficients of the composites surpassed that of LDPE and appeared to have increased with time.

However, LDPE/CS_6 exhibited greater rigidity at asperities, withstanding plastic deformation and asperity fracture. Nonetheless, Figure 8b–d indicates that even LDPE/CS_6 began to gradually succumb to the enormous $\sigma_{T}$ after ~500 s.

**Figure 9:**
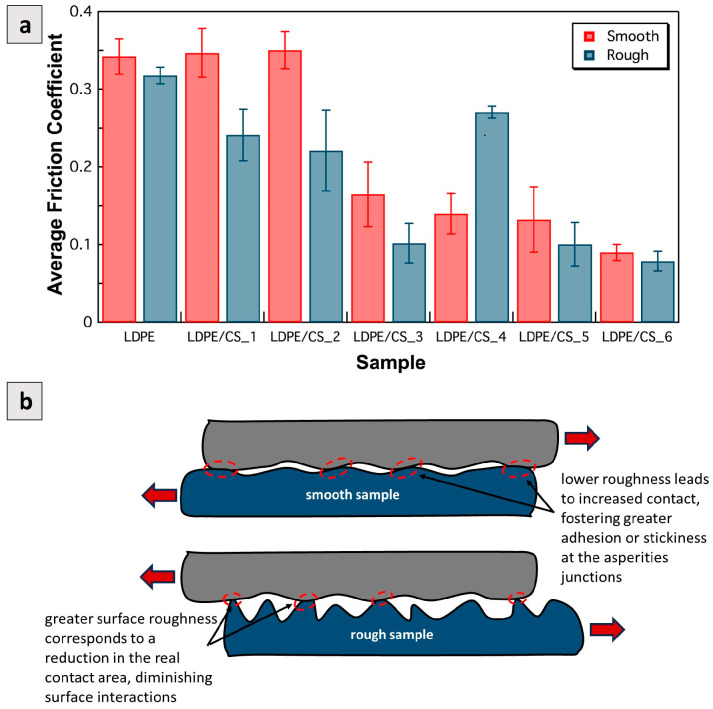
(**a**) Comparison of average friction coefficients of smooth vs. rough surfaces for LDPE samples with various CS loadings. The applied load was 5 N and the shear velocity was 10 RPM (8.4 mm/s); (**b**) schematic illustrating the relationship between surface roughness and surface interactions (adhesion) between polymer samples.

Figure 9a shows the average friction coefficients for the samples tested under 5 N and 10 RPM. In other words, these are discrete values derived from averaging the continuous values of Figure 7a and Figure 8a. A close examination of Figure 9a indicates that the friction coefficient of rough samples tended to be lower than that of smooth samples with equivalent particle loading [48]. This trend is in contrast to that of metals, which exhibit a higher coefficient of friction with higher roughness. In metals, the increased irregularities on the surface can cause interlockings and a higher resistance to the relative movement of the surfaces. Conversely, for polymers the adhesion at asperity contact points is very strong. When the surface roughness is reduced to only a few nanometers, the true area of contact (A_R_) is increased and the adhesion or stickiness increases, resulting in higher friction values. However, with more roughness the total area of contact is decreased, resulting in lesser surface interactions and lower friction (see Figure 9b). Indeed, the explanation provided may not universally apply to all composite systems. For instance, Soni et al. observed a higher wear rate with increased surface roughness in their study [17]. It is worth noting that the sizes of the filler combinations they utilized were several orders of magnitude greater than the carbon spheres (CSs) employed in this study. Furthermore, the concentration applied was significantly higher at 15 wt.% for each of the two fillers used (30 wt.% in total). This substantial particle size and concentration could potentially account for the high wear reported, as large particles would adhere loosely and easily get debonded. However, it is important to highlight that friction changes due to surface roughness were not reported in their study. 

The results from the friction experiments indicated that LDPE/CS_6 was consistently the best performing sample across varying roughness, load, and speed conditions. Compared to virgin LDPE, LDPE/CS_6 presents an optimal reduction in the friction coefficient of approximately 74% and 75% for smooth and rough samples, respectively. This value far exceeds those reported in the literature for LDPE composites under dry sliding conditions [19,24], thus illustrating the superiority of the LDPE/CS composite system. The enhancement in performance can be credited to the presence of CSs in this sample. The CS content effectively interrupts surface adhesion and enhances the mechanical properties of the composite, enabling it to withstand both normal and shear forces. Consequently, this increases its resistance to asperity deformation and adhesive failure. 

#### 3.4.2. Wear Study on LDPE/CS Composites

**Figure 10:**
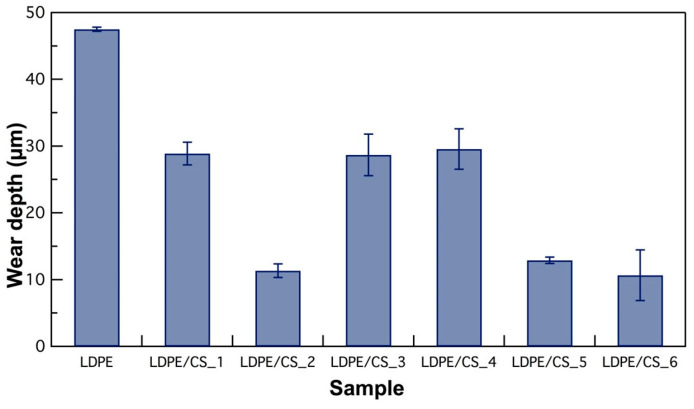
Plot of wear depth for LDPE and the different composite samples. Error bars are derived from the standard deviation of 3+ measurements along the wear track.

Following the wear runs, the wear tracks were profiled. Details of these profiles and a description of the wear mechanism evaluated using optical microscopy are found in Figure S5. The wear depth or height loss of the samples as measured by a confocal microscope is presented in Figure 10. The results reveal that the virgin LDPE sample exhibited the highest wear depth. Conversely, the wear depth decreased in the composites due to the load-bearing capabilities of CSs and the mechanical enhancements brought about by the particles such as improved hardness, rendering the composite more resistant to deformation and wear. 

However, the LDPE/CS_3 and LDPE/CS_4 samples displayed an unexpected increase in wear depth. This could be attributed to the occurrence of multiple three-body abrasions (in addition to other existing wear mechanisms), where particles from debris acted as third friction bodies, causing cuts and ploughing of the material. Moreover, it is possible that the particle contents in these samples were not sufficient at the surface to prevent penetration of the asperities of the counter pin. With increased CS loading (LDPE/CS_5 and LDPE/CS_6), the surface had a better distribution of particles such that interparticle distances may have been reduced, resulting in an improved ability to withstand high load. Further studies will be required to completely understand this phenomenon. CS loading improves surface hardness and hinders deformation and ploughing mechanisms. Consequently, wear depth decreased in LDPE/CS_5 and LDPE/CS_6 composites. Impressively, LDPE/CS_6 showcased a remarkable wear reduction of approximately 78% compared to virgin LDPE, establishing it as the superior composite. Again, this reduction in wear is greater than values reported in the literature for LDPE composite systems [19,24]. Figure S4 shows pictures of 3D optical wear profiles of samples after the wear test, as characterized by a confocal microscope.

## 4. Conclusions

Low-density polyethylene (LDPE) and varying amounts of carbon spheres (CSs) ranging from 0.5 to 8 wt.% were blended to create LDPE/CS composites through a simple casting method. The morphological, structural, thermal, mechanical, and tribological characteristics of the prepared composites were evaluated in this study. The following conclusions can be drawn from this research:

▪ Analysis through scanning electron microscopy and Raman spectroscopy confirmed the presence of CSs in LDPE, while X-ray diffraction results indicated that the intrinsic structure of LDPE remained unaffected by the addition of CSs.
▪ Thermal assessments from differential scanning calorimetry and thermogravimetric analysis revealed enhanced thermal properties in the composites. The crystallinity increased marginally with CS content up to 1 wt.%, suggesting that they acted as nucleation sites for crystallization. However, with increased particle content crystallinity reduces, possibly due to the particles inhibiting crystal growth and chain mobility. In addition, the composites demonstrated improved thermal stability owing to the strong interactions between particles and polymer, which slows down degradation.
▪ The mechanical tests revealed notable enhancements in the Young’s modulus, with improvements of up to 24%, and Shore D hardness, showing increases of up to 4% in the composites. These findings suggest the effective dispersion and interfacial interaction of the hard carbon spheres (CSs) with LDPE, resulting in a stiffer composite material.
▪ Comprehensive friction and wear studies were conducted under various conditions, including load, speed, and sample surface roughness, to discern the impact of these parameters and particle concentration on the friction coefficient and wear resistance. Surfaces with higher roughness were found to reduce friction due to decreased adhesive forces resulting from a smaller contact area. On the other hand, elevated load and speed increased resultant stress on surface asperities.
▪ The advantages of incorporating carbon sphere (CS) fillers became apparent in composites with a particle concentration of 1 wt.% and higher. This was attributed to the interruption of interactions at adhesive junctions, along with the reinforcing properties of the particles, enabling them to withstand deformation and resist fatigue and adhesive failure at asperities.
▪ The LDPE/CS_6 sample, with a CS loading of 8 wt.%, outperformed other composites in terms of friction values, representing a remarkable 75% reduction and exhibiting the lowest wear depth, indicating a 78% reduction compared to pristine LDPE. These findings suggest the potential of LDPE/CS composites for applications requiring robust, low-friction, and wear-resistant materials, particularly in biomedical and automotive sectors. This composite can also prove to be exceptionally valuable in dry condition applications, such as in space exploration. Additionally, the insights into the forces and mechanisms behind friction and wear reduction can guide the design of other polymer composites for diverse operating conditions.

## Figures and Tables

**Table 1:** Sample nomenclature and their corresponding carbon sphere (CS) content.

Sample Name	CS Loading (wt.%)
LDPE	0
LDPE/CS_1	0.25
LDPE/CS_2	0.5
LDPE/CS_3	1
LDPE/CS_4	2
LDPE/CS_5	4
LDPE/CS_6	8

**Table 2:** Test conditions for the tribology experiments.

Test Type	Top Surface Material	Normal Load (N)	Rotational Speed (RPM)	Sample Surface Roughness, Ra (nm)	Time (h)
Friction		5	10	1670	0.25
		5	100	1670	0.25
		20	10	1670	0.25
	HDPE	20	100	1670	0.25
		5	10	181	0.25
		5	100	181	0.25
		20	10	181	0.25
		20	100	181	0.25
Wear	SS	50	100	181	0.25

## Data Availability

All data are contained within the article and in the supporting documents. Raw data are also available only on request.

## Supplementary Materials

The following supporting information can be downloaded at: https://www.mdpi.com/article/10.3390/ma17071536/s1, Figure S1: SEM images showing the top, middle, and bottom cross-sectional view of (a) LDPE/CS_1 composite and (b) LDPE/CS_5 composite. The images confirm that the distribution of particles is not affected by zone refining. Figure S2: (a) XRD of CSs showing amorphous carbon represented by the broad peaks of (002) and (100) planes; (b) XRD plots of LDPE and all LDPE/CS composite samples. The XRD plot reveals two sharp distinct peaks at approximately 2Ɵ equal to 21.5° and 23.8°, representing the crystalline portion of LDPE. CS peaks are not seen in these spectra, possibly because of the low concentration of CSs in the polymer. Figure S3: Optical image of LDPE/CS_6 after friction experiments at high loads (20 N/100 RPM; R_a_ = 181 nm) at time (a) 0 s, (b) 300 s, (c) 700 s. These images show how the surface is removed gradually with time, thereby creating debris that induces more friction. Additionally, the true contact area increases and there is more surface contact for interaction. Figure S4: Optical image of wear area on (a) LDPE, (b) LDPE/CS_1, (c) LDPE/CS_2, (d) LDPE/CS_3, (e) LDPE/CS_4, (f) LDPE/CS_5, (g) LDPE/CS_6. Figure S5: 3D wear profile of (a) LDPE, (b) LDPE/CS_1, (c) LDPE/CS_2, (d) LDPE/CS_3, (e) LDPE/CS_4, (f) LDPE/CS_5, (g) LDPE/CS_6. Table S1: Thermal properties of samples (obtained from DSC heating and cooling scans). Table S2: Critical TGA parameters of samples. Table S3: Mechanical properties of samples derived from the tensile tests.
