# Supplementary material for: Enhancing the Tribological Properties of Low-Density Polyethylene Using Hard Carbon Microfillers
Source: Materials (Basel). 2024 Mar 28;17(7):1536. doi: 10.3390/ma17071536 (PMC11012580; doi:10.3390/ma17071536)
Supplement: Supplementary file 1 [file materials-17-01536-s001.zip › materials-2919155-supplementary.pdf]

SUPPORTING INFORMATION

Enhancing the tribological properties of low-density polyethylene using hard carbon microfillers

*Samuel E. Solomon [1], Rachel Hall [2], Jibao He [3], Vijay T. John [1] and Noshir S. Pesika [1,]**

[1] Department of Chemical and Biomolecular Engineering, Tulane University, 6823 St. Charles Ave., New Orleans, LA 70118. (USA)

[2] New Product Development, Intralox LLC, 301 Plantation Rd., New Orleans, LA 70123, USA

[3] Microscopy Laboratory, Tulane University, New Orleans, LA 70118, USA

*To whom correspondence should be addressed

Email: npesika@tulane.edu

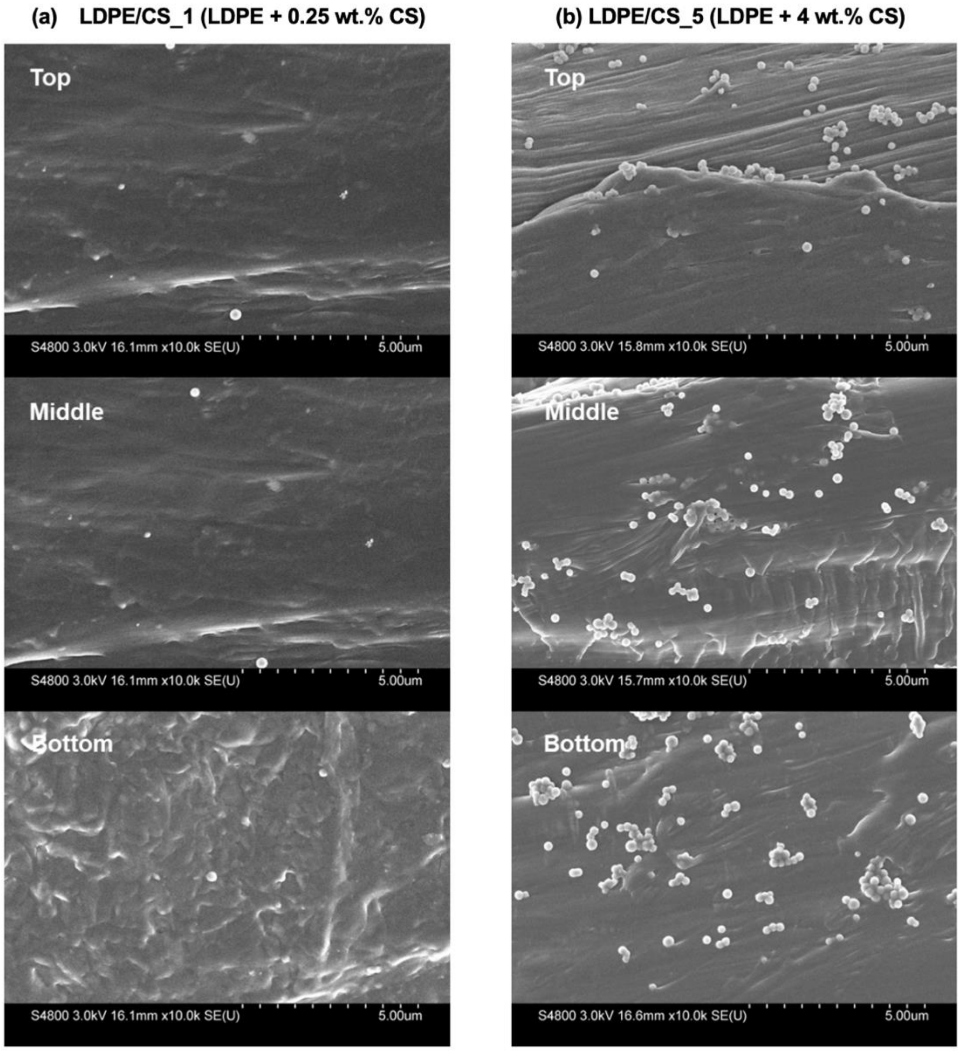

Figure S1: SEM images showing the top, middle and bottom cross-sectional view of (a) LDPE/CS_1 composite and (b) LDPE/CS_5 composite. The images confirm that the distribution of particles is not affected by zone refining.

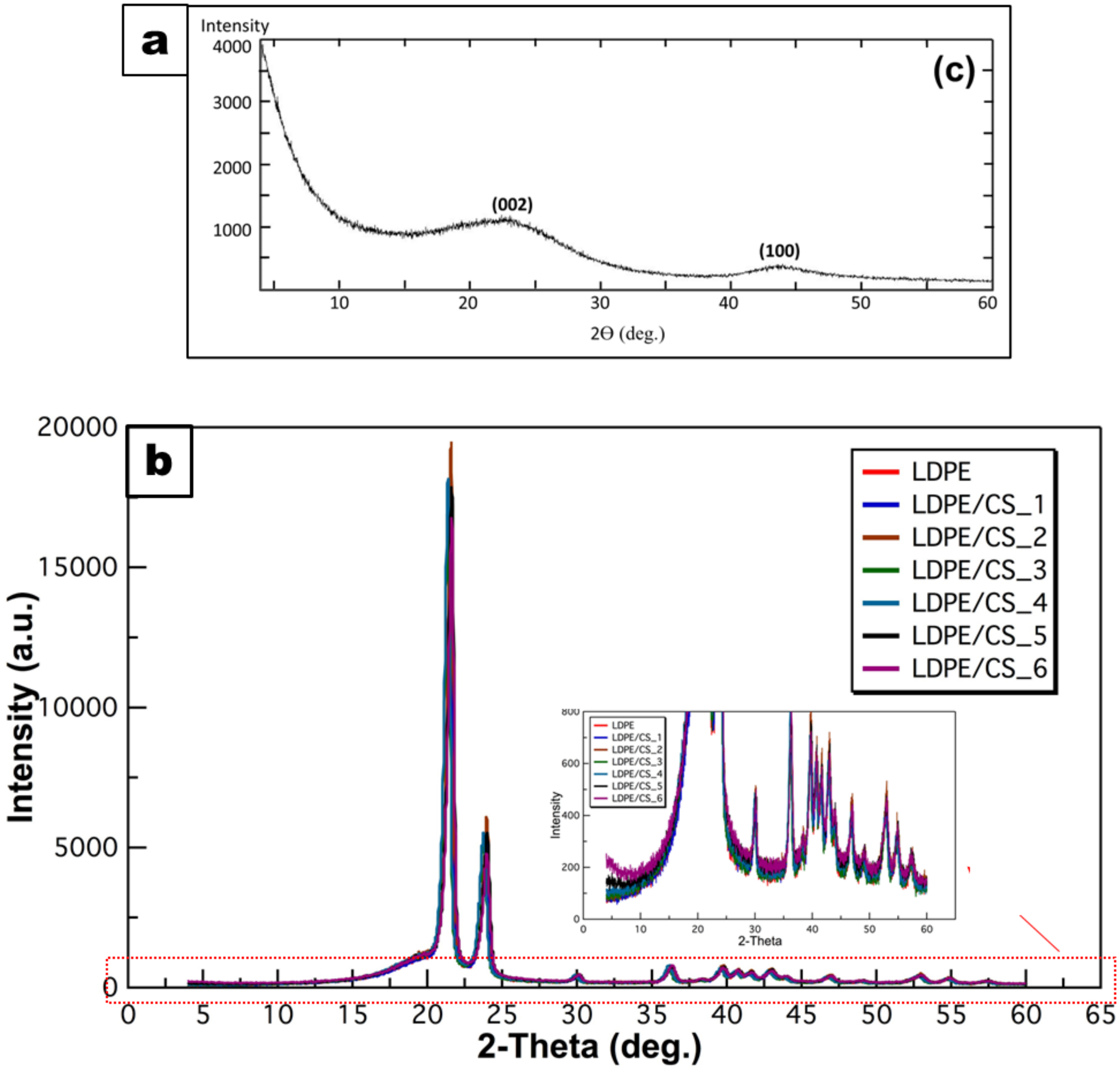

Figure S2: (a) XRD of CS showing amorphous carbon represented by the broad peaks of (002) and (100) planes (b) XRD plots of LDPE and all LDPE/CS composite samples. The XRD plot reveals two sharp distinct peaks at approximately 2Θ equal to 21.5° and 23.8°, representing the crystalline portion of LDPE. CS peaks are not seen in these spectra which may suggest that CS does not alter the polymer structure or possibly because of the low concentration of CS in the polymer.

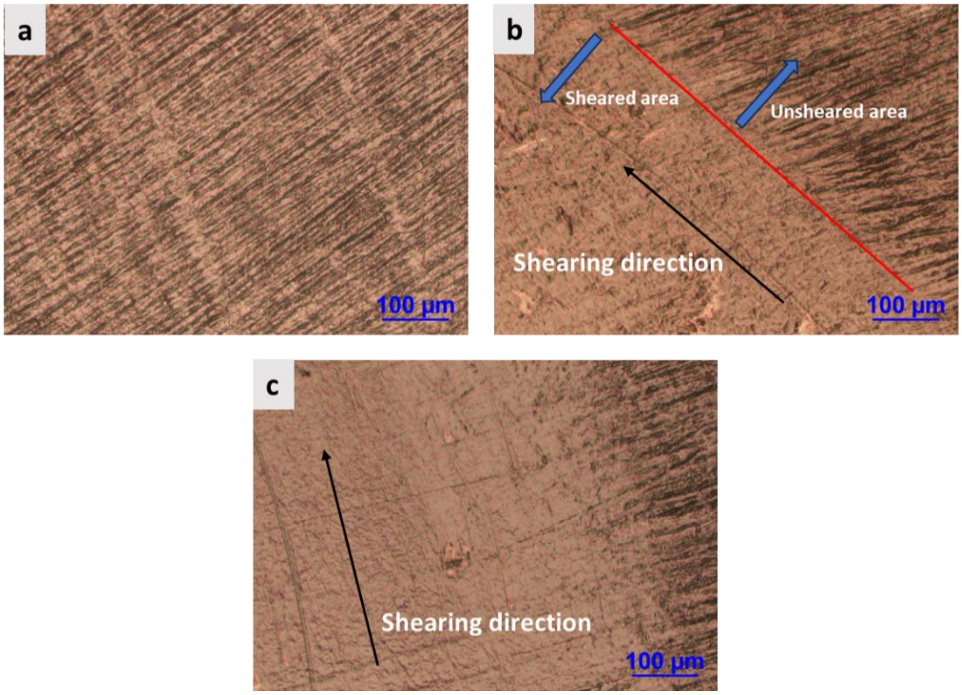

Figure S3: Optical image of LDPE/CS_6 after friction experiments at high loads (20 N/100 RPM; R_a = 181 nm) at time (a) 0 s (b) 300 s (c) 700 s. These images show how the surface is removed gradually with time thereby creating debris that induces more friction. Additionally, the true contact area increases and there is more surface contact for interaction.

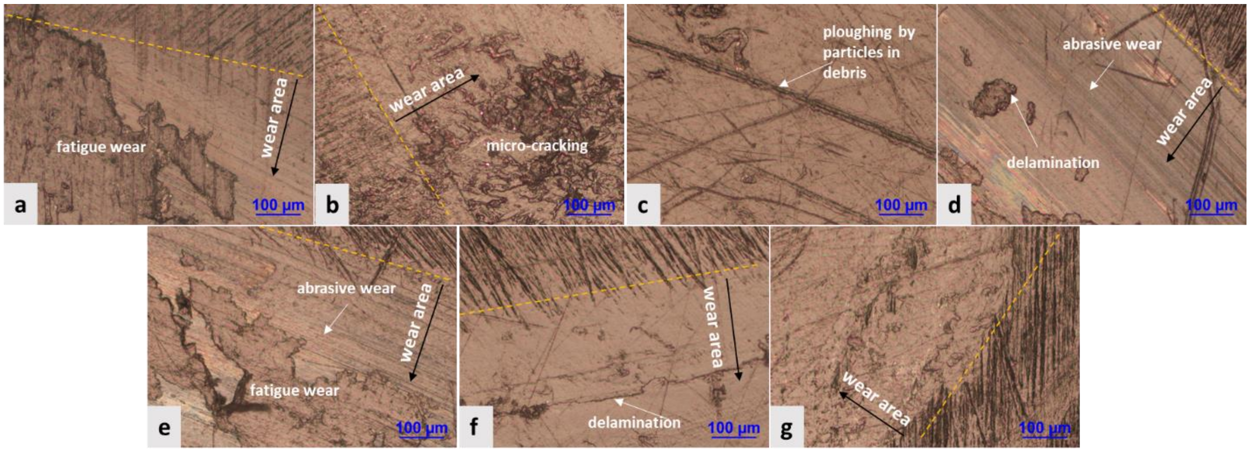

Figure S4: Optical image of wear area on (a) LDPE (b) LDPE/CS_1 (c) LDPE/CS_2 (d) LDPE/CS_3 (e) LDPE/CS_4 (f) LDPE/CS_5 (g) LDPE/CS_6. Identified wear mechanisms

include abrasive wear, fatigue wear, micro-cracking, ploughing from debonded particles, and potential delamination (see Figure S4). While all samples exhibited some form of abrasive wear due to the penetration of the stronger stainless steel (SS) into the polymer, a few differences were observed. In the LDPE sample (Figure S4a), a two-body abrasion between the mating surfaces was mostly observed. However, in the composites, a three-body abrasion involving debonded particles between the mating surfaces led to a ploughing action, resulting in trenches as evident in LDPE/CS_2 (Figure S4c). Fatigue wear and delamination were also commonly observed mechanisms, indicative of cyclic loading and load-induced micro-cracks, potentially leading to material separation and delamination.

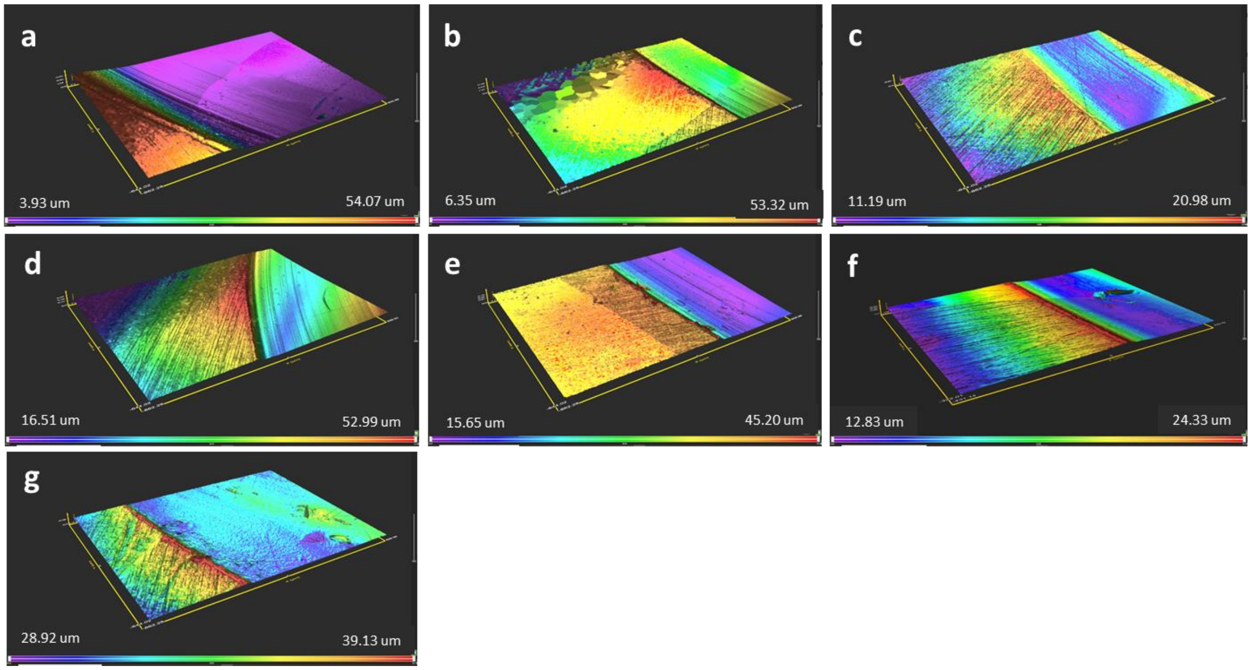

Figure S5. 3D wear profile of (a) LDPE (b) LDPE/CS_1 (c) LDPE/CS_2 (d) LDPE/CS_3 (e) LDPE/CS_4 (f) LDPE/CS_5 (g) LDPE/CS_6

Table S1: Thermal properties of samples (obtained from DSC heating and cooling scans)

Sample	Peak T_m (^{0}C)	Peak T_c (^{0}C)	Onset T_m (^{0}C)	Onset T_c (^{0}C)	ΔH_m (J/g)	ΔH_c (J/g)
LDPE	130.95±0.64	117.85±0	124.97±0.07	119.17±0	190.6± 6.9	176.8±3.2
LDPE/CS_1	130.30±0.15	118.64±0.13	124.92±0.34	119.46±0	189.6±2.8	172.8±2.4
LDPE/CS_2	130.25±0.23	118.57±0	124.92±0.39	119.47±0	196.5±7.4	177. 1 ±2.4
LDPE/CS_3	130.40±0.18	118.71±0	124.95±0.48	119.59±0	205. 3 ±10.8	186.1±5.4
LDPE/CS_4	130.30±0.12	118.57±0	124.42±0.30	119.56±0.01	189.9±7.9	171.7±6.6
LDPE/CS_5	130.65±0	118.77±0.01	124.66±0.03	119.67±0.01	172.9±7.9	155.8±8.9
LDPE/CS_6	130.22±0	118.86±0.07	124.67±0.03	119.87±0	172.1±7.0	158. 3 ±6.0

Table S2: Critical TGA parameters of samples

Sample	Onset point (^{0}C)	End point (^{0}C)	Onset degradation temp (Td_5) (^{0}C)	Temp. at 50% weight loss (Td_{50}) (^{0}C)
LDPE	419.1	448	391.25	433.7
LDPE/CS_1	422.4	450.6	405.4	435.5
LDPE/CS_2	422.1	450.9	400.5	437.1
LDPE/CS_3	422.8	451.8	405.7	437.5
LDPE/CS_4	422.9	450.3	402.1	436.8
LDPE/CS_5	423.7	451.2	404.6	438.4
LDPE/CS_6	421.8	450.8	404.4	437.5

Table S3: Mechanical properties of samples derived from the tensile tests.

Sample	Young's Modulus (GPa)	Stress* at maximum force (MPa)	Strain at maximum force (%)
LDPE	1.50±0.10	20.0±0.5	3.89±1.28
LDPE/CS_1	1.55±0	18.7±1.2	4.53±1.09
LDPE/CS_2	1.58±0.29	15.68±5.12	2.29±1.28
LDPE/CS_3	1.66±0.01	18.0±2.1	2.73±0.89
LDPE/CS_4	1.71±0.07	17.4±0.5	2.28±0.04
LDPE/CS_5	1.83±0.05	19.9±0.1	2.68±0.29
LDPE/CS_6	1.86±0.21	17.38±4.19	2.27±1.13

*Stress is engineering stress
